# Expected Hierarchical Integration Reduces Perceptions of a Low Status Group as Less Competent than a High Status Group While Maintaining the Same Level of Perception of Warmth

**DOI:** 10.3389/fpsyg.2016.02068

**Published:** 2017-01-09

**Authors:** Jianning Dang, Li Liu, Yuan Liang, Deyun Ren

**Affiliations:** Beijing Key Laboratory of Applied Experimental Psychology, School of Psychology, Beijing Normal UniversityBeijing, China

**Keywords:** compensation, social status, permeability, legitimacy, intergroup perception, stereotype

## Abstract

The compensation effect, namely people’s tendency to judge one group more positively on some dimensions and the other group more positively on other dimensions, has been validated using real social categories and experimentally created groups. However, less attention has been paid to whether and how changes in social structure affect the emergence of the compensation effect. The present research first replicated the compensation effect using Chinese participants (Study 1). Then, two studies were conducted to examine the effects of group boundary permeability (Study 2) and the legitimacy of the social hierarchy (Study 3) on the emergence of the compensation effect. The results demonstrated that the compensation effect was more likely to emerge when the group boundary was impermeable and when the social hierarchy was legitimate. The implications of these findings and the effect of social change on intergroup perception are discussed.

## Introduction

In the last few decades, the compensation effect has received much attention from social psychologists. From the intergroup process perspective, the compensation effect refers to the phenomenon that people judge the ingroup more positively on some dimensions and the outgroup more positively on other dimensions (e.g., Kervyn et al., 2009a,b, 2011). For example, Whites perceive Blacks as warmer but less competent, while Blacks perceive Whites as more competent but colder (Biernat et al., 2009). Most previous studies on the compensation effect have been conducted from a static perspective; that is, to validate the effect across different countries (Yzerbyt et al., 2005; Kervyn et al., 2008) and races (Biernat et al., 2009). However, it remains unknown whether and how social structural change affects the emergence of the compensation effect. Inspired by the social identity tradition, which focuses on the role of social context in intergroup relations (Reicher, 2004), the present research adopted a social dynamic perspective to explore the compensation effect. The aim was to examine the effects of group boundary permeability and the legitimacy of social hierarchy, two notable aspects of social structural change (Florack et al., 2003; Zhang et al., 2014), on the occurrence of the compensation effect.

### The Compensation Effect in Two Groups with Different Social Status

The compensation effect (Kervyn et al., 2010) mainly occurs in relation to two fundamental dimensions of social judgment (i.e., warmth and competence, Fiske et al., 2002, 2007; Cuddy et al., 2007, 2008). Previous research suggests that the compensation effect exists among groups with different social status (Yzerbyt et al., 2005; Biernat et al., 2009). Specifically, when two groups are judged, high social status group members are more likely to be viewed as more competent but colder, whereas members of the low-status group are viewed as warmer but less competent. Most importantly, this tendency to appraise two groups with different social status in a compensatory way reflects a high degree of consensus across high- and low-status group members (Jost et al., 2005; Yzerbyt et al., 2005). For example, one study showed that both French (high-status group) and Belgian (low-status group) participants associated French individuals with high levels of competence and low levels of warmth and associated Belgian individuals with low levels of competence and high levels of warmth (Yzerbyt et al., 2005).

### The Effects of Social Structure on the Compensation Effect

Following the traditional social identity approach, compensation has been interpreted as a social creativity strategy to re-establish positive social identity for low-status groups and as a magnanimity strategy to maintain positive social identity for high-status groups (Yzerbyt et al., 2008). When an ingroup’s low social status is unlikely to change, disadvantaged group members seek to bolster their standing on other comparative dimensions, although they have to acknowledge their ingroup inferiority on status-related dimensions (i.e., the social creativity strategy, van Knippenberg, 1978; Tajfel and Turner, 1986; Niens and Cairns, 2003). Members of an advantaged group whose superior status is fully ensured may attempt to appear non-discriminatory (Cambon et al., 2015) by showing no ingroup favoritism (Verkuyten and Reijerse, 2008) or even outgroup bias on such status-irrelevant dimensions as communion or warmth (Bettencourt et al., 2001; Leach et al., 2002; Vanbeselaere et al., 2006).

The above analysis indicates that compensation is a motivational strategy to maintain positive social identity when the status of advantaged and disadvantage groups is relatively stable. Therefore, we assumed that the emergence of compensation might rely on specific sociostructural conditions, and when the social structure changes, the compensation effect may disappear. Several empirical studies support our assumption. Terry (2003) finds that the social creativity and magnanimity strategies are usually adopted when the social structure is relatively unchangeable. Meanwhile, Cambon et al. (2015) find that perceived status legitimacy accounts to the indirect relationship between increased status difference and compensation effect. Specifically, the more asymmetrical social hierarchy is, the more legitimate people perceive their status and the more compensation they exhibit. However, this work was correlational in nature and failed to reveal causal relationship (Fiedler et al., 2011). Therefore, present research aimed to add to these literatures by exploring the effect of social structure on the compensation effect.

Given that group boundary permeability and the legitimacy of social hierarchy are key characteristics of social structure and closely related to stability of group status (Tajfel, 1978), the present research aimed to examine their effects on compensation. Permeability indicates the extent to which group members can leave one group and join another (Tajfel and Turner, 1986; Verkuyten and Reijerse, 2008). Legitimacy represents the extent to which privileged status is based on fair grounds (Tajfel, 1978). These two important characteristics determine people’s preferred responses to social hierarchy (Blair and Jost, 2003; Rubin et al., 2014; LeBlanc et al., 2015). Although there is no direct evidence of the effects of group boundary permeability and social hierarchy legitimacy on compensation, previous research regarding their influences on intergroup relations and identity management strategies informs our hypotheses.

### The Effect of Group Boundary Permeability on Compensation

Based on previous work, we predicted that high- and low-status group members would exhibit compensation when the group boundary was impermeable. On the one hand, the slim possibility of social mobility ensures the distinctive superiority of advantaged group members on status-relevant dimensions; therefore, they may show outgroup bias on status-irrelevant dimensions to manifest their magnanimity (Vanbeselaere et al., 2006). On the other hand, members of a disadvantaged group may re-establish positive social images for the ingroup and themselves by maintaining an advantage on comparative status-irrelevant dimensions while acknowledging ingroup inferiority on status-relative dimensions (Niens and Cairns, 2003) when they have no opportunities to change their group memberships. Therefore, it is likely that both high- and low-status group members will exhibit compensation when the group boundaries are impermeable.

In contrast, permeable group boundaries may elicit ingroup favoritism by advantaged group members and outgroup favoritism by disadvantaged group members on both status-relevant and status-irrelevant dimensions. One previous study finds that an open group boundary exposes threats to high-status group members’ positive distinctiveness; this motivates them to defend their group boundary and affirm their absolute superiority by differentiating themselves positively from disadvantaged group members (Scheepers and Ellemers, 2005). Moreover, permeable group boundaries also provide opportunities for low-status group members to change their group memberships; therefore, they may try to leave the disadvantaged ingroup and exhibit favoritism toward the advantaged outgroup (Niens and Cairns, 2003).

### The Effect of Legitimacy of Social Hierarchy on Compensation

In addition to the effects of an impermeable group boundary, we expected that a legitimate social hierarchy may facilitate groups with different social status to engage in compensation. Specifically, when the social hierarchy is legitimate, members of advantaged groups need not resort to maintaining their undisputed privileged position (LeBlanc et al., 2015). Therefore, they may treat the disadvantaged outgroup in a relatively fair or even generous way (Leach et al., 2002; Nadler et al., 2009; Spears et al., 2010) to relieve strong normative pressures (Cambon et al., 2015). The legitimate social status quo justifies disadvantaged group members’ inferiority on status-relevant dimensions (Mullen et al., 1992; Bettencourt et al., 2001). Therefore, to re-establish the ingroup’s positive social image, disadvantaged group members are motivated to enhance the ingroup on the status-irrelevant dimensions that best define their identity (Tajfel and Turner, 1986). Based on this reasoning, we hypothesized that compensation would occur when the social hierarchy was legitimate.

The compensation effect will no longer exist when the social hierarchy is perceived as illegitimate. Because people’s perception of competence derives from the social status of groups (Fiske et al., 2002), the competence superiority of so-called high-status group members will no longer be acknowledged when the social status quo is illegitimate. Moreover, an illegitimate social hierarchy motivates disadvantaged group members to elevate the status of the ingroup to match that of high-status outgroups (Rubin et al., 2014) or even to exhibit ingroup favoritism in an instrumental way to reverse the intergroup status hierarchy and obtain a prestigious position (Bettencourt et al., 2001; Niens and Cairns, 2003; Scheepers et al., 2009). In addition, when the social hierarchy is perceived as illegitimate, advantaged group members may feel shameful and guilty about their exhibition of ingroup favoritism on status-relevant dimensions, so they are likely to exhibit more positive outgroup evaluation and egalitarianism (Shepherd et al., 2013a,b) to make them feel better. Some research (Kuang and Liu, 2012) has revealed that advantaged group members showed less discrimination to disadvantaged group members when they were told that the social hierarchy status quo was illegitimate (versus legitimate).

### Overview of Present Research

The present research was conducted with Chinese participants. In China, citizens are divided into either a rural or an urban category according to a spatial hierarchy. This categorization is based on the *Hukou* system, a register of the location of individuals’ permanent residences (Chan and Buckingham, 2008). People in the rural category live in the countryside and make their living by farming; those in the urban category live in cities and enjoy life-long benefits provided by the government. Therefore, in terms of social-economic status and social reputation, the urban category is seen as superior to the rural category (Zhang et al., 2014). In recent years, the *Hukou* system has been reformed to facilitate social integration and these changes have challenged the legitimacy of the existing social hierarchy (Kuang and Liu, 2012) and facilitated rural-to-urban mobility (Zhang et al., 2014). This current social situation provides a natural laboratory within which to explore the influences of legitimacy and permeability on compensation in high (i.e., urban category) and low (i.e., rural category) social status group members.

To examine the influence of social structure change on the compensation effect, three studies were conducted. As there is no previous research on the compensation effect with Chinese participants, it was first necessary to replicate the compensation effect in two categories of Chinese citizens (Study 1). We hypothesized that both high- (urban) and low-status (rural) group members would perceive the ingroup and outgroup in a compensatory way; that is, the urban group would be viewed as more competent than the rural group, whereas the rural group would be viewed as warmer than the urban group (Hypothesis 1). Then we examined how group boundary permeability (Study 2) and legitimacy of social status (Study 3) affected the compensation effect. According to the analysis above, both high- and low-status members should engage in compensation when the group boundary is impermeable (Hypothesis 2) and the social hierarchy is legitimate (Hypothesis 3).

## Study 1: The Compensation Effect in Chinese Citizens

The aim of Study 1 was to examine whether the compensation effect was validated for Chinese individuals in the rural and urban categories. Compensation was measured using positive and negative attributive trait ratings (Fiske et al., 2002). As the urban group possessed greater social status, it was hypothesized that participants of both urban and rural groups would perceive individuals in the urban group as more competent but colder, whereas individuals in the rural group would be perceived as warmer but less competent.

### Materials and Methods

#### Ethics Statement

All the studies were reviewed and approved by the Committee of Protection of Subjects at Beijing Normal University. Before each study, all participants provided written informed consent and were debriefed at the end of the research according to the established committee guidelines.

#### Participants

Participants were 57 undergraduates (16 males and 41 females, aged from 18 to 23 years, *M* = 20.07, *SD* = 1.41) from a large Chinese university. Participants were asked to state their *Hukou* registration (i.e., rural or urban). Students who had lived in rural areas prior to their university enrollment were categorized as rural (*n* = 29); all such participants had lived in rural areas for at least 16 years. Students who had been living in cities were categorized as urban (*n* = 28).

#### Materials

##### Trait rating

Both urban and rural individuals were rated on 20 traits (Yzerbyt et al., 2005, 2008; Kervyn et al., 2009a): five positive-warmth traits (warm, nice, caring, tolerant, and sincere), five negative-warmth traits (cold, selfish, hostile, unfriendly, and irritable), five positive-competence traits (capable, conscientious, motivated, intelligent, and skilled) and five negative-competence traits (lazy, negligent, unintelligent, self-abased, and incompetent). Each trait was rated on a 7-point scale from 1 (*not at all*) to 7 (*totally*). Separate warmth and competence scores for the urban and rural groups (Cronbach’s alpha of the four scores >0.74) were computed by averaging the ratings of positive traits and the reversed ratings of negative traits on each dimension.

#### Procedure

Participants were informed that they were going to perform a social judgment task. After confirming that they understood the task purpose and procedure, participants were seated individually in front of a computer and completed the onscreen task. Before the task began, participants were asked to indicate their gender, age, and *Hukou* category (rural or urban). Then, participants completed the trait-rating task. The order in which participants rated urban and rural individuals first was counterbalanced and all the traits were presented randomly.

### Results and Discussion

A *post hoc* power analysis conducted using GPower (Faul et al., 2007) indicated that the achieved power of the compensation effect approached to 100% in this study, suggesting the sample size was large enough.

We conducted a 2 (Judge category: urban, rural) × 2 (Target category: urban, rural) × 2 (Dimension: warmth, competence) mixed-model analysis of variance (ANOVA) with the first factor varying between participants to analyze the compensation effect. A significant main effect of Target category showed that the rural group (*M* = 4.16, *SD* = 0.35) were evaluated more positively on general ratings than the urban group (*M* = 3.98, *SD* = 0.24), *F*(1,55) = 7.68, *p* = 0.008, η^2^ = 0.12. We also found a significant effect of Dimension, *F*(1,55) = 28.23, *p* < 0.001, η^2^ = 0.34, indicating that warmth ratings (*M* = 3.93, *SD* = 0.27) were generally lower than competence ratings (*M* = 4.20, *SD* = 0.28).

**FIGURE 1:**
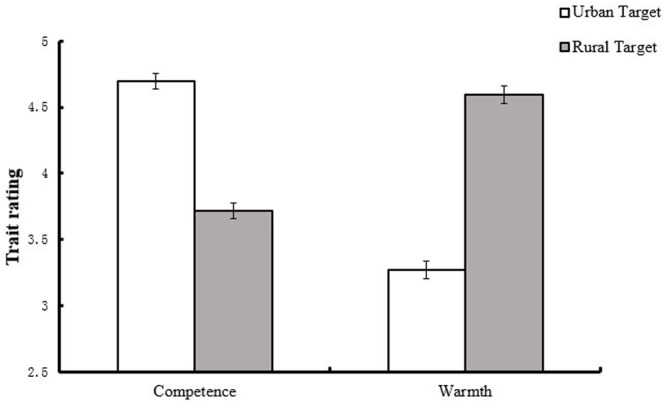
**Evaluation of warmth and competence as a function of target and judge.** Error bars represent standard error of means.

Most importantly, the expected Target category by Dimension interaction was significant, *F*(1,54) = 206.32, *p* < 0.001, η^2^ = 0.79. More competence was attributed to individuals in the urban group (*M* = 4.69, *SD* = 0.46) than to those in the rural group (*M* = 3.72, *SD* = 0.48), *t*(56) = 9.88, *p* < 0.001, Cohen’s *d* = 2.08. In contrast, more warmth was attributed to individuals in the rural group (*M* = 4.60, *SD* = 0.53) than to those in the urban group (*M* = 3.27, *SD* = 0.45), *t*(56) = 12.14, *p* < 0.001, Cohen’s *d* = 2.71 (see **Figure 1**). Meanwhile, urban target was rated higher on competence than on warmth [*t*(56) = 13.95, *p* < 0.001, Cohen’s *d* = 3.12], while rural target was rated higher on warmth than on competence [*t*(56) = 9.22, *p* < 0.001, Cohen’s *d* = 1.74]. Thus, the Target category by Dimension interaction demonstrated the emergence of compensatory pattern of social perception.

The present study replicated previous findings and tested whether the response patterns observed by Yzerbyt et al. (2008) and Kervyn et al. (2016) emerged in Chinese participants. Study 1 revealed the existence of compensation among Chinese participants; specifically, both high-status (urban) and low-status (rural) participants perceived advantaged group members as more competent but colder than disadvantaged group members. These results were consistent with previous findings (Jost et al., 2005; Biernat et al., 2009) and suggested that compensatory stereotypes were acknowledged across high- and low-status groups.

## Study 2: The Effect Of Group Boundary Permeability On Compensation

Study 1 confirmed the existence of the compensation effect among Chinese urban and rural participants. Study 2 was designed to investigate whether the compensation effect was affected by group boundary permeability. We hypothesized that high- and low-status group members would exhibit compensation when the group boundary was impermeable.

### Materials and Methods

#### Participants

Participants were 129 college students (55 males) aged from 17 to 22 years (*M* = 19.50, *SD* = 1.05) from a Chinese university. Participants were asked to indicate their *Hukou* registration (rural or urban). Students who lived in rural areas prior to their university enrollment were categorized as rural (*n* = 65); all such participants had lived in rural areas for at least 16 years. Students who had been living in cities were categorized as urban (*n* = 64). Participants were randomly divided into a permeable (*n* = 68) or an impermeable condition (*n* = 61). Across the two experimental conditions, rural and urban participants were fairly and evenly distributed, χ^2^ = 0.07, *p* = 0.795.

#### Materials

##### Manipulation of group boundary permeability

Participants were asked to read an article (Zhang et al., 2014, see in Appendix) ostensibly excerpted from newspapers. The article given to participants in the permeable condition stated that increasing numbers of rural residents were moving to cities through several routes, such as education or career (see Appendix 1). The article given to participants in the impermeable condition stated that rural residents faced many difficulties when they tried to move to the city (see Appendix 2). Both articles were of the same length.

After reading the article, participants responded to several questions, including a manipulation check and items about readability of the article (i.e., “This article is easy to understand.” “The meaning of this article is clear”). The item “In principle, it is not difficult for a rural resident to be considered as an urban resident,” adapted from the group boundary permeability scale developed by Mummendey et al. (1999), was used to check the effectiveness of the manipulation. Responses to this item were on a 7-point scale ranging from 1 (*completely disagree*) to 7 (*completely agree*); higher scores indicated greater permeability.

##### Trait rating

The same traits used in Study 1 were employed in Study 2. Separate warmth and competence scores for the urban and rural groups were computed by averaging the ratings of positive traits and the reversed ratings of negative traits on each dimension (Cronbach’s alpha for the four scores >0.75).

#### Procedure

Participants were informed to finish a reading task and a social judgment task after they provided informed consent. First, Participants were asked to read an article which was used to manipulate the group boundary. Then, participants were asked to complete the same trait-rating task used in Study 1 to measure compensation; in this task, urban and rural individuals were rated on positive and negative warmth- and competence-related traits. The order in which participants rated urban and rural categories was counterbalanced and all the traits were presented randomly.

### Results and Discussion

A *post hoc* power analysis indicated that the achieved power of the compensation effect approached to 100% with the sample size of 129 in this study, suggesting the sample size was large enough.

#### Manipulation Check

We submitted participants’ ratings of group boundary permeability to a 2 (Condition: permeable, impermeable) × 2 (Judge category: urban, rural) ANOVA, which showed that only the main effect of Condition was significant, *F*(1,125) = 10.73, *p* < 0.001, η^2^ = 0.08, suggesting that participants in the permeable condition (*M* = 5.69, *SD* = 1.46) reported greater perceived permeability than those in the impermeable condition (*M* = 4.74, *SD* = 1.89). Thus, the manipulation of boundary permeability was successful.

#### Effect of Permeability on the Compensation Effect

To explore the effect of permeability on compensation, a 2 (Condition: permeable, impermeable) × 2 (Judge category: urban, rural) × 2 (Dimension: warmth, competence) × 2 (Target category: urban, rural) mixed-model ANOVA of trait ratings was conducted, in which the first two variables were between-subject factors and the latter two were within-subject factors. Three main effects were found: for Target category, *F*(1,125) = 61.67, *p* < 0.001, η^2^ = 0.33; Dimension, *F*(1,125) = 23.74, *p* < 0.001, η^2^ = 0.16; and Condition, *F*(1,125) = 7.71, *p* = 0.006, η^2^ = 0.06. The rural group (*M* = 4.84, *SD* = 0.66) was evaluated more positively on general ratings than the urban group (*M* = 4.39, *SD* = 0.46). The competence dimension (*M* = 4.70, *SD* = 0.55) was rated higher than the warmth dimension (*M* = 4.53, *SD* = 0.48). Participants in the permeable condition (*M* = 4.72, *SD* = 0.42) gave higher general ratings than those in the impermeable condition (*M* = 4.49, *SD* = 0.49).

A two-way interaction emerged between Dimension and Target category, *F*(1,125) = 152.14, *p* < 0.001, η^2^ = 0.55. Simple effect analysis suggested that the rural group (*M* = 5.05, *SD* = 0.77) was perceived as warmer than the urban group (*M* = 4.01, *SD* = 0.62), *t*(128) = 11.58, *p* < 0.001, Cohen’s *d* = 1.49, whereas the urban group (*M* = 4.78, *SD* = 0.61) was perceived as more competent than the rural group (*M* = 4.63, *SD* = 0.64), *t*(128) = 2.78, *p* = 0.006, Cohen’s *d* = 0.24. Meanwhile, urban target was rated higher on competence than on warmth [*t*(128) = 10.68, *p* < 0.001, Cohen’s *d* = 1.25], while rural target was rated higher on warmth than on competence [*t*(128) = 8.88, *p* < 0.001, Cohen’s *d* = 0.59].

**FIGURE 2:**
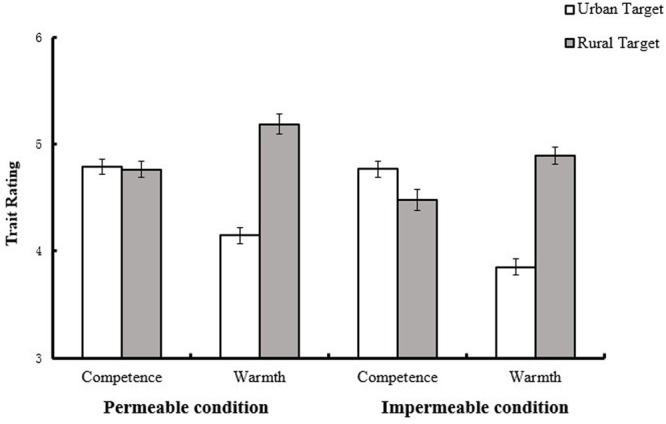
**Participants’ ratings of urban and rural groups in different conditions (Study 2).** Error bars represent standard error of means.

Moreover, simple main effects analyses revealed that, the rural group was perceived as warmer than the urban group in both conditions, *M*_rural_ = 5.19, *SD*_rural_ = 0.70, *M*_urban_ = 4.15, *SD*_urban_ = 0.63, *F*(1,125) = 69.59, *p* < 0.001, η^2^ = 0.36 for the permeable condition, and *M*_rural_ = 4.89, *SD*_rural_ = 0.81, *M*_urban_ = 3.85, *SD*_urban_ = 0.58, *F*(1,125) = 62.15, *p* < 0.001, η^2^ = 0.33 for the impermeable condition. However, the urban group (*M* = 4.77, *SD* = 0.67) was perceived as more competent than the rural group (*M* = 4.48, *SD* = 0.65), but only in the impermeable condition, *F*(1,125) = 13.65, *p* < 0.001, η^2^ = 0.10, and when the group boundary was permeable, both the urban group (*M* = 4.79, *SD* = 0.55) and the rural group (*M* = 4.76, *SD* = 0.61) were perceived as equally competent, *F*(1,125) = 0.13, *p* = 0.725, η^2^ = 0.001 (see **Figure 2**). Therefore, although the omnibus Condition × Dimension × Target category interaction was not so significant [*F*(1,125) = 1.74, *p* = 0.189, η^2^ = 0.01], the data supported Hypothesis 2.

The absence of the four-way interaction [*F*(1,125) = 0.20, *p* = 0.675, η^2^ = 0.002] indicated that the effect of group boundary permeability on compensation among rural and urban participants was not moderated by the category of judges. Further analysis (see **Table 1**) revealed, rural judges rated ingroup warmer [*F*(1,125) = 37.02, *p* < 0.001, η^2^ = 0.23] and less competent [*F*(1,125) = 4.30, *p* = 0.04, η^2^ = 0.03] than outgroup under impermeable condition, while they rated ingroup warmer [*F*(1,125) = 36.52, *p* < 0.001, η^2^ = 0.23] than but equally competent [*F*(1,125) = 2.18, *p* = 0.143, η^2^ = 0.02] as outgroup under permeable condition. For urban judges, they rated ingroup colder [*F*(1,125) = 25.56, *p* < 0.001, η^2^ = 0.17] and more competent [*F*(1,125) = 9.99, *p* = 0.002, η^2^ = 0.07] than outgroup under impermeable condition, while they rated ingroup colder [*F*(1,125) = 33.17, *p* < 0.001, η^2^ = 0.21] than but equally competent [*F*(1,125) = 0.89, *p* = 0.349, η^2^ = 0.007] as outgroup under permeable condition. In conclusion, the compensation effect vanished for both rural and urban judges under permeable condition.

**Table 1:** Rural and Urban participants’ perception of ingroup and outgroup under different conditions (Study 2).

		Impermeable condition		Permeable condition	
		Warmth	Competence	Warmth	Competence
Rural Judge	Ingroup	4.98 (0.79)	4.49 (0.60)	5.28 (0.61)	4.87 (0.70)
	Outgroup	3.83 (0.47)	4.72 (0.64)	4.22 (0.74)	5.02 (0.52)
Urban Judge	Ingroup	3.87 (0.67)	4.81 (0.70)	4.15 (0.63)	4.55 (0.49)
	Outgroup	4.81 (0.84)	4.47 (0.71)	5.10 (0.79)	4.64 (0.49)

In Study 2, the effect of group boundary permeability on compensation was examined and the results suggested that, regardless of the category of the judge, high- and low-status group members were perceived in a compensatory way when the group boundary was impermeable. These findings provide evidence for the noblesse oblige effect (Vanbeselaere et al., 2006) that advantaged group members express reduced favoritism toward the ingroup when they possess absolute control over the ingroup, and when there is no possibility (or a remote possibility) for outgroup members to join them. Moreover, low-status members view compensation as a social creativity strategy (Niens and Cairns, 2003) to re-establish positive social identity; this is more likely to occur when the group boundary is impermeable (Verkuyten and Reijerse, 2008).

Unfortunately, some findings were contrary to our hypothesis. We found that when the group boundary was permeable, disadvantaged group members exhibited ingroup favoritism by minimizing intergroup differences in competence and maintaining their priority on the status-irrelevant dimension (i.e., warmth), whereas advantaged group members did not overemphasize their advantage on competence but acknowledged low-status members’ superiority on warmth. We suggest explanations for these unexpected results in the general discussion.

## Study 3: The Effect of Legitimacy of Social Hierarchy on Compensation

Study 3 was designed to investigate the effect of the legitimacy of the social status quo on compensation. We hypothesized that both high-status (urban) and low-status (rural) individuals would exhibit more compensation when the social hierarchy was legitimate.

### Materials and Methods

#### Participants

Participants were 120 college students (42 male) aged between 17 and 24 years (*M* = 19.38, *SD* = 1.51) from a Chinese university. Participants were asked to indicate their *Hukou* registration (rural or urban). Students who lived in rural areas prior to their university enrollment were categorized as rural (*n* = 61); all such participants had lived in rural areas for at least 16 years. Students who had been living in cities were categorized as urban (*n* = 59). Participants were randomly divided into a legitimate (*n* = 61) or an illegitimate condition (*n* = 59). Across the two experimental conditions, rural and urban participants were fairly evenly distributed, χ^2^ = 1.21, *p* = 0.272.

#### Materials

##### Manipulation of legitimacy of social hierarchy

Participants were asked to read an article (Kuang and Liu, 2012, see in Appendix) ostensibly excerpted from newspapers. The article in the legitimate condition explained that the *Hukou* system, which categorizes Chinese citizens as either urban or rural, was legitimate and would not be abolished in the future (see Appendix 3). The article in the illegitimate condition stated that the *Hukou* system was illegitimate and would be abolished in the future (see Appendix 4). Both articles were of the same length.

After reading the article, participants responded to several questions, including a manipulation check item and items about readability of the article used in Study 2. We used the item “Rural residents can demand to be as well off as urban residents,” adapted from a previous study (Mummendey et al., 1999), to measure participants’ perception of the legitimacy of the status quo after they read the article. The item was reverse-scored and higher scores indicated higher levels of legitimacy.

##### Trait rating

The same traits used in Study 1 and Study 2 were employed in Study 3. Separate warmth and competence scores for the urban and rural groups were computed by averaging the ratings of positive traits and the reversed ratings of negative traits on each dimension (Cronbach’s alpha for the four scores >0.79).

#### Procedure

Participants were informed to finish a reading task and a social judgment task after they provided informed consent. First, participants were asked to read an article which was used to manipulate the legitimacy of social hierarchy. Then, participants were asked to complete the same trait-rating task used in Study 1 and 2 to measure compensation; in this task, urban and rural individuals were rated on positive and negative warmth- and competence-related traits. The order in which participants rated urban and rural categories was counterbalanced and all the traits were presented randomly.

### Results and Discussion

A *post hoc* power analysis indicated that the achieved power of the compensation effect approached to 100% with the sample size of 120 in this study, suggesting the sample size was large enough.

#### Manipulation Check

A 2 (Condition: legitimate, illegitimate) × 2 (Judge category: urban, rural) ANOVA was conducted on participants’ perceptions of legitimacy. Only the main effect of Condition was significant, *F*(1,116) = 97.13, *p* < 0.001, η^2^ = 0.46, indicating that participants in the legitimate condition (*M* = 4.36, *SD* = 1.13) reported higher levels of legitimacy than those in the illegitimate condition (*M* = 2.49, *SD* = 0.90). Thus, the legitimacy manipulation was successful.

#### Effect of Legitimacy on the Compensation Effect

A 2 (Condition: legitimacy, illegitimacy) × 2 (Judge category: urban, rural) × 2 (Target category: urban, rural) × 2 (Dimension: warmth, competence) mixed-model ANOVA of trait ratings was conducted to explore the effect of the legitimacy manipulation on compensation; the first two variables were between-subject and the latter two were within-subject factors. Three main effects were found: for Target category, *F*(1,116) = 61.35, *p* < 0.001, η^2^ = 0.35; Dimension, *F*(1,116) = 27.71, *p* < 0.001, η^2^ = 0.19; and Judge category, *F*(1,116) = 5.44, *p* = 0.021, η^2^ = 0.05. The rural group (*M* = 4.96, *SD* = 0.76) was evaluated more positively on general ratings than the urban group (*M* = 4.40, *SD* = 0.63). The competence dimension (*M* = 4.78, *SD* = 0.61) was rated higher than the warmth dimension (*M* = 4.57, *SD* = 0.63). Rural participants (*M* = 4.79, *SD* = 0.61) gave higher ratings than urban participants (*M* = 4.56, *SD* = 0.51).

A two-way interaction emerged between Dimension and Target category, *F*(1,116) = 135.99, *p* < 0.001, η^2^ = 0.54. Follow-up analyses suggested that the rural group (*M* = 5.16, *SD* = 0.85) was perceived as warmer than the urban group (*M* = 3.98, *SD* = 0.83), *t*(119) = 11.45, *p* < 0.001, Cohen’s *d* = 1.41, whereas the urban group (*M* = 4.82, *SD* = 0.71) was perceived to be as competent as the rural group (*M* = 4.75, *SD* = 0.77), *t*(119) = 0.91, *p* = 0.360, Cohen’s *d* = 0.10. Meanwhile, the urban group was rated higher on competence than on warmth [*t*(119) = 10.40, *p* < 0.001, Cohen’s *d* = 1.09], while the rural group was rated higher on warmth than on competence [*t*(119) = 8.36, *p* < 0.001, Cohen’s *d* = 0.51].

**FIGURE 3:**
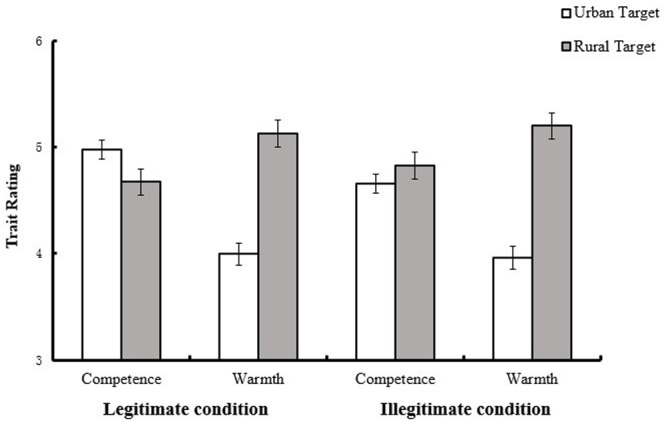
**Participants’ ratings of urban and rural groups in different conditions (Study 3).** Error bars represent standard error of means.

The expected Target × Dimension × Condition interaction was marginally significant, *F*(1,116) = 3.72, *p* = 0.056, η^2^ = 0.03. Simple main effects analyses revealed that, when the social hierarchy was legitimate, the rural group (*M* = 5.13, *SD* = 0.82) was perceived as warmer than the urban group (*M* = 4.00, *SD* = 0.85), *F*(1,116) = 62.71, *p* < 0.001, η^2^ = 0.35 whereas the urban group (*M* = 4.97, *SD* = 0.68) was perceived as more competent than the rural group (*M* = 4.67, *SD* = 0.72), *F*(1,116) = 8.28, *p* = 0.005, η^2^ = 0.07. When the social hierarchy was illegitimate, the rural group (*M* = 5.20, *SD* = 0.89) was perceived as warmer than the urban group (*M* = 3.96, *SD* = 0.81), *F*(1,116) = 67.84, *p* < 0.001, η^2^ = 0.37, whereas the urban group (*M* = 4.66, *SD* = 0.72) was *not* perceived as more competent than the rural group (*M* = 4.83, *SD* = 0.81), *F*(1,116) = 3.21, *p* = 0.076, η^2^ = 0.03 (see **Figure 3**).

The absence of the four-way interaction [*F*(1,116) = 0.46, *p* = 0.498, η^2^ = 0.004] indicated that the effect of social hierarchy legitimacy on compensation among rural and urban participants was not moderated by the category of the judge. Further analysis (see **Table 2**) revealed that, for rural judges, they rated ingroup warmer [*F*(1,116) = 41.29, *p* < 0.001, η^2^ = 0.26] and slightly less competent [*F*(1,116) = 3.01, *p* = 0.085, η^2^ = 0.03] than outgroup under legitimate condition, while they rated ingroup warmer [*F*(1,116) = 48.97, *p* < 0.001, η^2^ = 0.30] than but equally competent [*F*(1,116) = 2.18, *p* = 0.757, η^2^ = 0.001] as outgroup under illegitimate condition. For urban judges, they rated ingroup colder [*F*(1,116) = 22.20, *p* < 0.001, η^2^ = 0.16] and more competent [*F*(1,116) = 6.82, *p* = 0.01, η^2^ = 0.06] than outgroup under legitimate condition, while they rated ingroup colder [*F*(1,116) = 23.06, *p* < 0.001, η^2^ = 0.17] and even less competent [*F*(1,116) = 4.49, *p* = 0.036, η^2^ = 0.04] than outgroup under illegitimate condition. In conclusion, the compensation effect vanished for both rural and urban judges under illegitimate condition.

**Table 2:** Rural and Urban participants’ perception of ingroup and outgroup under different conditions (Study 3).

		Legitimate condition		Illegitimate condition	
		Warmth	Competence	Warmth	Competence
Rural Judge	Ingroup	5.53 (0.82)	4.86 (0.77)	5.29 (0.88)	4.79 (0.82)
	Outgroup	4.16 (1.05)	5.12 (0.75)	3.91 (0.76)	5.74 (0.64)
Urban Judge	Ingroup	3.86 (0.61)	4.85 (0.60)	4.02 (0.88)	4.55 (0.80)
	Outgroup	4.78 (0.64)	4.49 (0.55)	5.09 (0.91)	4.88 (0.80)

Study 3 explored the effect of legitimacy on compensation. The results supported the hypothesis that both high- and low-status group members would manifest compensatory stereotypes when they were primed to perceive the hierarchy as legitimate. These results complement the finding of Cambon et al. (2015) that perceived legitimacy is positively associated with compensation. The pressure toward non-discrimination forced advantaged group members to compensate the low-status outgroup on the warmth dimension (Cambon et al., 2015). In contrast, disadvantaged group members were more likely to re-establish a positive social image for the ingroup by making social comparisons on the dimension that best defines themselves (i.e., warmth) when they had to admit the superiority of the outgroup on the status-defining dimension (i.e., competence; Niens and Cairns, 2003).

Moreover, in accord with previous studies, when the social hierarchy was illegitimate, disadvantaged group members adopted a social competition strategy (Niens and Cairns, 2003); that is, they rejected the superiority of the high-status group and sought positive distinctiveness for the ingroup on the status-defining dimension, to strive for equal or even superior social status for the ingroup. In contrast, advantaged group members acknowledged the disadvantaged outgroup’s superiority on warmth but did *not* continue to claim their advantage on competence. This result can be understood in terms of the advantaged group’s emotional and behavioral reactions to intergroup inequality. When the relatively higher status of the ingroup is perceived as illegitimate, advantaged group members may experience shame toward the ingroup and sympathy toward the disadvantaged outgroup; these group-based emotions prevent them from displaying ingroup favoritism (Shepherd et al., 2013a,b).

## General Discussion

The present research verified the compensation effect among Chinese participants and explored the sociostructural conditions of the emergence of compensation. Study 1 replicated the compensation effect with two real groups of different social status categorized by the *Hukou* system. The manipulation of group boundary permeability and social hierarchy legitimacy revealed, respectively, a compensation effect when the intergroup boundary was impermeable (Study 2) and when the social hierarchy was legitimate (Study 3).

### Theoretical and Practical Contributions of Present Research

The present research adds to existing research in several ways. First, most previous studies have only validated the existence of compensation (e.g., Kervyn et al., 2008; Biernat et al., 2009) and our findings further suggest that compensation does not always persist. The manipulation of social structure factors in the present research revealed that group boundary permeability and social hierarchy legitimacy are situational factors that affect compensation. Second, the present research combined a social identity perspective with stereotype content and provided evidence for the effect of social structure changes on strategies to manage identity. The expression of compensation reflected social creativity and magnanimity strategies to rebuild or maintain positive social identity for low-and high-status groups, respectively (Yzerbyt et al., 2008). The present research indicates that these strategies are adopted when the possibility of intergroup mobility is minimal or when the social hierarchy is considered legitimate. In contrast, the complementary pattern to evaluate each other on warmth and competence no longer existed if the group boundary was open or the legitimacy of social hierarchy was denied. These findings support the suggestion that social creativity and magnanimity strategies are used more readily under relatively unchangeable social conditions (Terry, 2003).

The present findings have some practical implications. In recent years, Chinese citizens have experienced the reform of the *Hukou* system. These reforms have challenged the fairness of the existing social hierarchy (Kuang and Liu, 2012) and facilitated rural-to-urban mobility (Zhang et al., 2014), and several tentative official policies have been tested in some places (Chan and Buckingham, 2008). The present research found that the salience of a permeable group boundary and illegitimate social hierarchy inhibited the occurrence of the relatively harmonious intergroup relations, (i.e., compensation) and increased disadvantaged group members’ favoritism toward the ingroup. However, these findings do not convince us of the infeasibility of the *Hukou* system reform or the desirability of maintaining an unfair status quo. The social changes resulting from the *Hukou* system reform have offered some advantages for low-status group members and decreased advantaged group members’ discrimination toward low-status group members (see Zhang et al., 2014). This dilemma requires the creation of more suitable ways to introduce social structure changes that do not arouse new prejudice and that allow shifts to the status quo satisfy everybody.

### Discussion of Several Findings Inconsistent with Previous Research

Findings of Study 2 indicated that the effect of a permeable group boundary on intergroup perception was more complex than we had expected. We found that disadvantaged group members exhibited ingroup favoritism on both dimensions when the group boundary was perceived as permeable. Although this contradicts some previous research (e.g., Lalonde and Silverman, 1993), there are also research indicating that permeable group boundary does not necessarily result in individual mobility among disadvantaged group members (e.g., Ellemers et al., 1992). Jackson (Jackson et al., 1996, Experiment 2) supposed that not all disadvantage group members but only those less identifiers choose to leave their ingroup when upward mobility is possible. So, when rural Chinese realize they can migrate to cities by their efforts, they may increase their confidence in ingroup capability and identification with ingroup, which makes them remain loyal to ingroup and take actions favorable to ingroup (Blair and Jost, 2003).

Meanwhile, our Study 2 results also showed that a permeable group boundary decreased rather than increased the advantaged group’s ingroup favoritism, which is contrary to some previous research (e.g., Scheepers and Ellemers, 2005). Agreeing with previous researchers (Zhang et al., 2014), we think this inconsistency may result from the different nature of group boundaries. The group boundary between rural and urban Chinese is mainly caused by geographical classification other than biological classification (e.g., skin color or race). The rural-to-urban mobility may increase the homogeneity between rural and urban Chinese and promote urban Chinese to perceive a more common ingroup identity with rural residence (Zhang et al., 2014), which then reduces prejudice and discrimination (Gaertner et al., 1993; Florack et al., 2003).

### Future Direction

Some issues need to be addressed in future studies. First, our participants were urban and rural undergraduates, so further research on other groups naturally differing in status is needed to examine whether our findings can be extended to other kinds of Chinese social groups. Second, as the accuracy of stereotype has attracted more and more attention, more research should be conducted to test whether what we found is the compensation effect or just accurate social perception about rural and urban Chinese. As proposed by Jussim et al. (2015), we can compare people’s descriptive beliefs about the groups to the objective criteria that establish group characteristics. We can also measure and compare rural and urban people’s motivation under different conditions or test whether the motivation mediates the relationship between social structure and the compensation effect. Third, the curvilinear relation between groups’ perceived competence and warmth (i.e., warmth peaks at average competence; Imhoff and Koch, in press; Koch et al., 2016) suggests that the compensation effect only applies to groups with high and average competence, but not to groups with high and low or average and low competence. Future research should examine this boundary condition of the compensation effect. Finally, future studies should use more concise and unequivocal essay manipulations of status permeability and illegitimacy to examine whether it is actually these two or other aspects of social structure that led to the breakdown of the compensation effect in the studies reported here.

## Author Contributions

JD contributed to all aspects of work for this article. LL contributed to conception, and design and revising the article critically. YL and DR contributed to data analysis and interpretation and revising the article critically.

## Conflict of Interest Statement

The authors declare that the research was conducted in the absence of any commercial or financial relationships that could be construed as a potential conflict of interest.

## References

[B1] Bettencourt B. A. Charlton K. Dorr N. Hume D. L. (2001). Status differences and in-group bias: a meta-analytic examination of the effects of status stability, status legitimacy, and group permeability. *Psychol. Bull.* 127 520–542. 10.1037/0033-2909.127.4.520 11439710

[B2] Biernat M. Sesko A. K. Amo R. B. (2009). Compensatory stereotyping in interracial encounters. *Group Process. Intergroup Relat.* 12 551–563. 10.1177/1368430209337469

[B3] Blair I. V. Jost J. T. (2003). Exit, loyalty, and collective action among workers in a simulated business environment: interactive effects of group identification and boundary permeability. *Soc. Justice Res.* 16 95–108. 10.1023/A:1024271019738

[B4] Cambon L. Yzerbyt V. Yakimova S. (2015). Compensation in intergroup relations: an investigation of its structural and strategic foundations. *Br. J. Soc. Psychol.* 54 140–158. 10.1111/bjso.12067 24660757

[B5] Chan K. Buckingham W. (2008). Is China abolishing the hukou system? *China Q.* 195 582–606.

[B6] Cuddy A. J. Fiske S. T. Glick P. (2007). The BIAS map: behaviors from intergroup affect and stereotypes. *J. Pers. Soc. Psychol.* 92 631–648. 10.1037/0022-3514.92.4.631 17469949

[B7] Cuddy A. J. Fiske S. T. Glick P. (2008). Warmth and competence as universal dimensions of social perception: The Stereotype Content Model and the BIAS map. *Adv. Exp. Soc. Psychol.* 40 61–149. 10.1016/S0065-2601(07)00002-0

[B8] Ellemers N. Doosje B. Van Knippenberg A. Wilke H. (1992). Status protection in high status minority groups. *Eur. J. Soc. Psychol.* 22 123–140. 10.1002/ejsp.2420220203

[B9] Faul F. Erdfelder E. Lang A.-G. Buchner A. (2007). GPower 3: a flexible statistical power analysis program for the social, behavioral, and biomedical sciences. *Behav. Res. Methods* 39 175–191. 10.3758/BF03193146 17695343

[B10] Fiedler K. Schott M. Meiser T. (2011). What mediation analysis can (not) do. *J. Exp. Soc. Psychol.* 47 1231–1236. 10.1016/j.jesp.2011.05.007

[B11] Fiske S. T. Cuddy A. J. Glick P. (2007). Universal dimensions of social cognition: warmth and competence. *Trends Cogn. Sci.* 11 77–83. 10.1016/j.tics.2006.11.005 17188552

[B12] Fiske S. T. Cuddy A. J. Glick P. Xu J. (2002). A model of (often mixed) stereotype content: competence and warmth respectively follow from perceived status and competition. *J. Pers. Soc. Psychol.* 82 878–902. 10.1037/0022-3514.82.6.878 12051578

[B13] Florack A. Piontkowski U. Rohmann A. Balzer T. Perzig S. (2003). Perceived intergroup threat and attitudes of host community members toward immigrant acculturation. *J. Soc. Psychol.* 143 633–648. 10.1080/00224540309598468 14609057

[B14] Gaertner S. L. Dovidio J. F. Anastasio P. A. Bachman B. A. Rust M. C. (1993). “The common ingroup identity model: recategorization and the reduction of intergroup bias,” in *European Review of Social Psychology* (Vol. 4) eds Stroebe W. Hewstone M. (Chichester: Wiley) 1–26.

[B15] Imhoff R. Koch A. (in press). How orthogonal are the big two of social perception? On the curvilinear relation between agency and communion. *Perspect. Psychol. Sci.* 10.1177/1745691616657334 28073333

[B16] Jackson L. A. Sullivan L. A. Harnish R. Hodge C. N. (1996). Achieving positive social identity: social mobility, social creativity, and permeability of group boundaries. *J. Pers. Soc. Psychol.* 70 241–254. 10.1037/0022-3514.70.2.241

[B17] Jost J. T. Kivetz Y. Rubini M. Guermandi G. Mosso C. (2005). System-justifying functions of complementary regional and ethnic stereotypes: cross-national evidence. *Soc. Justice Res.* 18 305–333. 10.1007/s11211-005-6827-z

[B18] Jussim L. Crawford J. T. Rubinstein R. S. (2015). Stereotype (in)accuracy in perceptions of groups and individuals. *Curr. Dir. Psychol. Sci.* 24 490–497. 10.1177/0963721415605257

[B19] Kervyn N. Bergsieker H. B. Grignard F. Yzerbyt V. Y. (2016). An advantage of appearing mean or lazy: amplified impressions of competence or warmth after mixed descriptions. *J. Exp. Soc. Psychol.* 62 17–23. 10.1016/j.jesp.2015.09.004

[B20] Kervyn N. Judd C. M. Yzerbyt V. Y. (2009a). You want to appear competent? Be mean! You want to appear sociable? Be lazy! Group differentiation and the compensation effect. *J. Exp. Soc. Psychol.* 45 363–367. 10.1016/j.jesp.2008.08.006

[B21] Kervyn N. Yzerbyt V. Y. Judd C. M. Nunes A. (2009b). A question of compensation: the social life of the fundamental dimensions of social perception. *J. Pers. Soc. Psychol.* 96 828–842. 10.1037/a0013320 19309205

[B22] Kervyn N. Yzerbyt V. Judd C. M. (2010). Compensation between warmth and competence: antecedents and consequences of a negative relation between the two fundamental dimensions of social perception. *Eur. Rev. Soc. Psychol.* 21 155–187. 10.1080/13546805.2010.517997

[B23] Kervyn N. Yzerbyt V. Y. Demoulin S. Judd C. M. (2008). Competence and warmth in context: the compensatory nature of stereotypic views of national groups. *Eur. J. Soc. Psychol.* 38 1175–1183. 10.1002/ejsp.526

[B24] Kervyn N. Yzerbyt V. Y. Judd C. M. (2011). When compensation guides inferences: indirect and implicit measures of the compensation effect. *Eur. J. Soc. Psychol.* 41 144–150. 10.1002/ejsp.748

[B25] Koch A. Imhoff R. Dotsch R. Unkelbach C. Alves H. (2016). The ABC of stereotypes about groups: agency/socioeconomic success, conservative–progressive beliefs, and communion. *J. Personal. Soc. Psychol.* 110 675–709. 10.1037/pspa0000046 27176773

[B26] Kuang L. Liu L. (2012). Prejudice against rural-to-urban migrants: the role of the hukou system in China. *PLoS ONE* 7:e46932 10.1371/journal.pone.0046932 PMC3489849 23144794

[B27] Lalonde R. N. Silverman R. A. (1993). Behavioral preferences in response to social injustice: the effects of group permeability and social identity salience. *J. Personal. Soc. Psychol.* 66 78–85. 10.1037/0022-3514.66.1.78

[B28] Leach C. W. Snider N. Iyer A. (2002). “”Poisoning the consciences of the fortunate”: the experience of relative advantage and support for social equality,” in *Relative Deprivation: Specification, Development and Integration* eds Walker I. Smith H. J. (New York, NY: Cambridge University Press) 136–163.

[B29] LeBlanc J. Beaton A. M. Walker I. (2015). The downside of being up: a new look at group relative gratification and traditional prejudice. *Soc. Justice Res.* 28 143–167. 10.1007/s11211-015-0233-y

[B30] Mullen B. Brown R. Smith C. (1992). Ingroup bias as a function of salience, relevance and status: an integration. *Eur. J. Soc. Psychol.* 22 103–122. 10.1002/ejsp.2420220202

[B31] Mummendey A. Klink A. Mielke R. Wenzel M. Blanz M. (1999). Socio-structural characteristics of intergroup relations and identity management strategies: results from a field study in East Germany. *Eur. J. Soc. Psychol.* 29 259–285. 10.1002/(SICI)1099-0992(199903/05)29:2/3<259::AID-EJSP927>3.0.CO;2-F

[B32] Nadler A. Harpaz-Gorodeisky G. Ben-David Y. (2009). Defensive helping: threat to group identity, ingroup identification, status stability, and common group identity as determinants of intergroup help-giving. *J. Pers. Soc. Psychol.* 97 823–834. 10.1037/a0015968 19857004

[B33] Niens U. Cairns E. (2003). Explaining social change and identity management strategies new directions for future research. *Theory Psychol.* 13 489–509. 10.1177/09593543030134003

[B34] Reicher S. (2004). The context of social identity: domination, resistance, and change. *Polit. Psychol.* 25 921–945. 10.1111/j.1467-9221.2004.00403.x

[B35] Rubin M. Badea C. Jetten J. (2014). Low status groups show in-group favoritism to compensate for their low status and compete for higher status. *Group Process Intergroup Relat.* 17 563–576. 10.1177/1368430213514122

[B36] Scheepers D. Ellemers N. (2005). When the pressure is up: the assessment of social identity threat in low and high status groups. *J. Exp. Soc. Psychol.* 41 192–200. 10.1016/j.jesp.2004.06.002

[B37] Scheepers D. Spears R. Manstead A. S. R. Doosje B. (2009). The influence of discrimination and fairness on collective self-esteem. *Personal. Soc. Psychol. Bull.* 35 506–515. 10.1177/0146167208329855 19139161

[B38] Shepherd L. Spears R. Manstead A. S. (2013a). ‘This will bring shame on our nation’: the role of anticipated group-based emotions on collective action. *J. Exp. Soc. Psychol.* 49 42–57. 10.1016/j.jesp.2012.07.011 23690650 PMC3657186

[B39] Shepherd L. Spears R. Manstead A. S. (2013b). When does anticipating group-based shame lead to lower ingroup favoritism? The role of status and status stability. *J. Exp. Soc. Psychol.* 49 334–343. 10.1016/j.jesp.2012.10.012

[B40] Spears R. Greenwood R. de Lemus S. Sweetman J. (2010). “Legitimacy, social identity and power,” in *The Social Psychology of Power* eds Guinote A. Vescio T. (New York, NY: Guilford Press) 251–283.

[B41] Tajfel H. (1978). “Individual and intergroup behavior,” in *Differentiation between Social Groups: Studies in the Social Psychology of Intergroup Relations* ed. Tajfel H. (San Diego, CA: Academic Press) 27–60.

[B42] Tajfel J. Turner J. C. (1986). “The Social Identity Theory of intergroup behavior,” in *Psychology of Intergroup Relations* eds Worchel S. Austin G. (Chicago, IL: Nelson-Hall) 7–24.

[B43] Terry D. J. (2003). “A social identity perspective on organizational mergers: the role of group status, permeability, and similarity,” in *Social Identity at Work: Developing Theory for Organizational Practice* eds Haslam A. S. van Knippenberg D. Platow M. J. Ellemers N. (New York, NY: Psychology Press) 223–240.

[B44] van Knippenberg A. (1978). “Status differences, comparative relevance, and intergroup differentiation,” in *Differentiation between Social Groups: Studies in the Social Psychology of Intergroup Relations* ed. Tajfel H. (New York, NY: Academic Press) 171–199.

[B45] Vanbeselaere N. Boen F. Van Avermaet E. Buelens H. (2006). The Janus face of power in intergroup contexts: a further exploration of the noblesse oblige effect. *J. Soc. Psychol.* 146 685–699. 10.3200/SOCP.146.6.685-699 17172145

[B46] Verkuyten M. Reijerse A. (2008). Intergroup structure and identity management among ethnic minority and majority groups: the interactive effects of perceived stability, legitimacy, and permeability. *Eur. J. Soc. Psychol.* 38 106–127. 10.1002/ejsp.395

[B47] Yzerbyt V. Provost V. Corneille O. (2005). Not competent but warm really? Compensatory stereotypes in the French-speaking world. *Group Process. Intergroup Relat.* 8 291–308. 10.1177/1368430205053944

[B48] Yzerbyt V. Y. Kervyn N. Judd C. M. (2008). Compensation versus halo: the unique relations between the fundamental dimensions of social judgment. *Personal. Soc. Psychol. Bull.* 34 1110–1123. 10.1177/0146167208318602 18593867

[B49] Zhang X. X. Zheng J. Liu L. Zhao X. Sun X. M. (2014). The effect of group boundary permeability on intergroup prejudice: the case of rural-to-urban migrants in China. *J. Pac. Rim Psychol.* 8 53–61. 10.1017/prp.2014.7

