# Supplementary material for: Expected Hierarchical Integration Reduces Perceptions of a Low Status Group as Less Competent than a High Status Group While Maintaining the Same Level of Perception of Warmth
Source: Front Psychol. 2017 Jan 9;7:2068. doi: 10.3389/fpsyg.2016.02068 (PMC5220094; doi:10.3389/fpsyg.2016.02068)
Supplement: Supplementary file 1 [file Data_Sheet_1.docx]

**Appendix**

**Appendix 1. Priming material of the permeability condition (Study 2)**

The household registration system (i.e., *Hukou* System) is a basic state administrative system. China's Hukou System divides citizens into agricultural (rural) and non-agricultural (urban) category. The current Hukou System does not completely isolate rural and urban people. At present, the mobility of urban and rural population gradually increased. More and more rural people enter the city through education, employment, buying houses in the city or some other methods. According to the surveys, billions of migrant workers work in new industrial areas, and tens of millions of white-collar workers who come from rural areas live in large cities. Therefore, rural people can enter the city through various means, and strive to get the same social benefits and treatment as urban people.

**Appendix 2. Priming material of the impermeability condition (Study 2)**

The household registration system (i.e., Hukou System) is a basic state administrative system. China's Hukou System divides citizens into agricultural (rural) and non-agricultural (urban) category. The Hukou System largely separates rural and urban populations from each other. Although some rural people try to enter the city through the means of education, employment, or buying houses in the city, they still face many problems, such as slim job opportunities and expensive housing. In addition, the living condition of migrant workers in the city is also more embarrassing. Their social status is relatively low and they still cannot enjoy the same social benefits and treatment as the urban people. Therefore, the general rural people’s dream to become a true urban citizen is still very difficult to achieve.

**Appendix 3. Priming material of the legitimacy condition (Study 3)**

The household registration system (i.e., Hukou System) is a basic state administrative system. China's Hukou System divides citizens into agricultural (rural) and non-agricultural (urban) category. The existing Hukou System has its own significances. First of all, the implementation of China's birth control policy dependents on Hukou System. According to the Hukou system, the children who are not registered into the rural household registration cannot enjoy the normal education and training, which to some extent can prevent "bounce" and limit the population growth. Second, the Hukou system restricts the influx of rural people who have no living skills in cities, and reduces the social resources spent on the resettlement of basic life necessities for these people. Therefore, although some people now call for the abolition of the existing household registration system, according to the current situation in China, immediately unifying urban and rural household registration will bring great difficulties in urban management, Therefore, the State Council announced the *Hukou* system would not be canceled in the short term.

**Appendix 4. Priming material of the illegitimacy condition (Study 3)**

The household registration system (i.e., *Hukou* System) is a basic state administrative system. China's *Hukou* System divides citizens into agricultural (rural) and non-agricultural (urban) category. The current *Hukou* system has become an obstacle to economic and social development and has become the basis for many discriminatory policies to be implemented. In terms of education, students in rural areas are facing unequal competition. For example, students with urban residence can enter universities more easily than rural students. In terms of employment, many institutions and companies tend to have the urban candidates when they recruit civil servants or employees. Hukou system also leads to different treatments of urban and rural residents in housing, social security and other aspects, which may increase the polarization of the rich and the poor. Therefore, many experts believe that the existing dual household registration system is unreasonable and it is an urgent need to reform it. In recent years, the State Council has gradually begun to develop a series of initiatives to abolish the existing household registration system.
